# Editorial: Acute promyelocytic leukemia - towards a chemotherapy-free approach to cure in all patients, Volume II

**DOI:** 10.3389/fonc.2023.1238486

**Published:** 2023-06-27

**Authors:** Harinder Gill, Nigel Russell, Yok-Lam Kwong

**Affiliations:** ^1^ Department of Medicine, School of Clinical Medicine, Li Ka Shing (LKS) Faculty of Medicine, the University of Hong Kong, Hong Kong, Hong Kong SAR, China; ^2^ Department of Haematology, Nottingham City Hospital and University of Nottingham, Nottingham, United Kingdom

**Keywords:** acute promyelocytic leukemia (APL), arsenic trioxide, oral arsenic trioxide, early death, epidemiology, chemotherapy-free

In the real-world setting, early death (ED) is an important factor compromising the outcome of newly-diagnosed acute promyelocytic leukemia (APL). In population-based studies of unselected patients with newly-diagnosed APL, ED rates of 10-60% were reported (1–14). The development of international recommendations for managing APL has led to a gradual improvement of ED with time, falling from 28% in the 1990s to approximately 15% in past two decades (14–16).. Risk factors for EDs included older age, high-risk disease, poor performance status and co-existing infections (17). Furthermore, factors that increased fatal hemorrhages, including high leucocyte count, elevated lactate dehydrogenase, low fibrinogen, impaired coagulation parameters and APL differentiation syndrome (APL-DS), also increased EDs (18–22). Delays in the administration of all-trans retinoic acid (ATRA) was also a major factor contributing to EDs at the community care level (22, 23). In large epidemiologic studies, delayed ATRA administration, leukocytosis and hemostatic abnormalities were major predictors for ED. In this volume, Wen et al. highlighted the contributions of Sanz low- and intermediate-risks and other clinical and hematologic parameters to EDs.

APL-DS is another important cause of mortality and morbidity in newly-diagnosed APL. Leukocytosis at presentation is a key predictor of APL-DS, which may be attenuated or prevented by the early use of chemotherapy. However, the impact chemotherapy-free induction with ATRA and arsenic trioxide (ATO) on the incidence, duration and sequelae of APL-DS is not well-defined. LaBella et al. retrospectively compared two cohorts of patients receiving ATRA/ATO with or without chemotherapy as induction therapy, with respect to changes in hematological parameters and the incidence and duration of APL-DS.

Less than 2% of patients with APL by morphology harbor gene fusion transcripts other than *PML::RARA*. These atypical fusion transcripts significantly impact on responses to ATRA and ATO. Guarnera et al. comprehensively reviewed acute myeloid leukemia (AML) with *RARA* rearrangements or rearrangements involving other members of the retinoic acid receptors including *RARB* and *RARG*. Ding et al. further described a case of AML with *HNRNPC::RARG* that morphologically mimicked APL. RNA sequencing is an important diagnostic tool for patients with AML driven by gene fusions. Liu et al. described the utility of RNA-sequencing in identifying novel fusions not detectable with conventional karyotyping, using AML with *FIP1L1::RARA* as an example.

With evolving therapeutic strategies, the demographics and epidemiology of APL are also changing. Ethnic differences in the incidences of APL are emerging, together with a shift in the peak age at presentation to the elderly (5, 14, 24). Furthermore, the curability of APL brings into focus the long-term safety of treatment, especially the development of second primary cancers (25, 26). Kumana et al. described how changes the introduction of oral-ATO-based regimens impacted on the epidemiology and prevalence of APL in Hong Kong. They further explored the potential repurposing of oral-ATO in other conditions, which included nucleophosmin-1 (*NPM1*)-mutated AML, multiple myeloma, mantle cell lymphoma, lung cancers, systemic lupus erythematosus, graft-versus-host disease and idiopathic pulmonary fibrosis (27–39).

To conclude this volume, Iyer et al. and Masetti et al. summarized the current treatment paradigms and future directions in the management of adult and pediatric patients with APL. The advent of ATO has significantly changed frontline protocols, with most induction regimens currently incorporating intravenous ATO with ATRA with or without chemotherapy (40–44), which have resulted in complete remission rates of 90–100% and long-term survivals of 86–97%. The role of oral-ATO formulated in Hong Kong has also emerged (45), and shown to be efficacious for APL in first relapse (R1), inducing second complete remission (CR2) in more than 90% of patients (46, 47). In the CR1 maintenance setting, oral-ATO-based regimens were safe and resulted in favorable survivals (48). Oral-ATO has been advanced into frontline protocols since 2013 with excellent long-term outcome (49, 50). In the real-world setting, oral-ATO-based induction in newly diagnosed APL reduced EDs, prevented relapses and improved overall survivals (51).

## Author contributions

HG conceived the Research Topic, wrote and approved the manuscript. NR and Y-LK co-edited the Research Topic and approved the manuscript. All authors contributed to the article and approved the submitted version.
